# Supplementary material for: Global wood fuel production estimates and implications
Source: Nat Commun. 2025 Jul 15;16:6227. doi: 10.1038/s41467-025-59733-y (PMC12264136; doi:10.1038/s41467-025-59733-y)
Supplement: Supplementary file 1 — Supplementary Information [file 41467_2025_59733_MOESM1_ESM.pdf]

Supplementary Information

Global wood fuel production estimates and implications

E. Ashley Steel[1*], Oliver Stoner[1,2], Harry Podschwit[1], Bruno Paz[3], Ilaria Bombelli[4,5], Sophia L. Simon[1,6], Erin Peterson[7], Holger Weimar[8], Sebastian Glasenapp[8], Richard Sikkema[9], Nazik Elhassan[10], Rob Bailis[11], Florian Steierer[12], Leonardo R. Souza[13]

[1]Forestry Division, Food and Agriculture Organization of the United Nations (FAO), Terme di Caracalla, Rome 00153.

[2]School of Mathematics and Statistics, University of Glasgow, 132 University Place, Glasgow, G12 8QQ, Scotland, UK.

[3]Department of Agricultural Economics and Rural Development, University of Göttingen, Göttingen, Germany

[4]Department of Statistical Sciences, Sapienza University of Rome, 00185, Rome, Italy.

[5]Current: Italian National Institute of Statistics, 00184, Rome, Italy.

[6]Current: GreenCollar. The ASN Co Building, 3 Hickson Road, The Rocks, NSW 2000, Australia.

[7]EP Consulting, Brisbane, QLD 4000, Australia

[8]Thünen Institute of Forestry, Leuschnerstrasse 91, 21031 Hamburg, Germany

[9]Tall Forester Trees (advisory services), Heteren, the Netherlands

[10]International Renewable Energy Agency (IRENA), Masdar City P.O. Box 236, Abu Dhabi, UAE

[11]Stockholm Environment Institute, Somerville, Massachusetts, USA

[12]United Nations Economic Commission for Europe, Geneva, Switzerland

[13]United Nations Statistics Division, 2 UN Plaza, 16th Floor, New York 10017 USA

*Address correspondence to: Ashley.Steel@FAO.org

Supplementary Methods 1: Data Preparation

Compilation of predictor variables and imputation of missing values

The set of potential predictors was derived from the conceptual model (as described in "Drivers of wood fuel demand and production – a conceptual model"), built via the expert working group (as described in "Expert group and development of the conceptual model"), and then screened with respect to availability of global data sources and completeness by country (and year if relevant). A few groups of variables represented redundant or similar concepts, e.g., Urban Population, Rural Population and Percent Urban, or Minimum Temperature and other metrics of temperature. From these groups, only the variables with the clearest mechanistic interpretation were retained, e.g., Percent Urban and Rural Population or Minimum Temperature (Supplementary Table 1).

For those potential predictors selected for modeling, approximately 30% of data were missing and required imputation. First, the percentage of people mainly using biomass or charcoal for cooking (Biomass Use, Charcoal Use) (Supplementary Table 1) were entirely missing for high-income countries and territories. Countries and territories were classified as high income by the World Bank for the 2024 fiscal year if they had a 2022 gross national income (GNI) per capita (calculated using the World Bank Atlas method) of $13,846 or above. As is current practice for tracking Sustainable Development Goal 7[1], we assumed Biomass Use and Charcoal Use both equal zero for these high-income countries .

Non-time varying predictors Minimum Temperature and Rainfall were only missing for the Falkland Islands (Malvinas). We assumed both were equal to the minimum values recorded for other countries or territories in the South America intermediate M49 region[2].

Time-varying predictors Forest Area and Wooded Area were imputed using linear-interpolation in countries or territories where the predictor in question was observed in at least two years. Other time-varying predictors Electricity, GDP, Gini Index, Life Expectancy, OIR, Population, Rural Population, Sawlogs, and Total Population were linearly interpolated in countries or territories where the predictor in question was observed in at least 75% of years between 1999-2019.

We filled the approximately 10% remaining missing covariate values using Multiple Imputation by Chained Equations (MICE) with Random Forests, implemented using the missForest function from the R package missForest v1.50[3]. The method was used to create 1000 imputed data sets, resulting in a distribution of imputed values for each missing data point. The mean over the 1000 data sets was chosen to produce a single set of imputed values. We carried out a qualitative assessment of uncertainty using Monte Carlo simulations and bootstrapped confidence intervals to determine whether substantial patterns of uncertainty in imputed values across space or time needed to be incorporated into the models.

We summarized imputation variability, for a given data point, by the coefficient of variation over the 1000 imputed data sets and further summarized these by computing the median of the coefficients of variability for each predictor variable and cluster (Supplementary Figure 1). By this measure, imputation variability was consistently high for OIR and Sawlogs, which also had relatively high levels of missingness before MICE (about 26% and 22%, respectively). Beyond this, we could not see a clear relationship between missingness and imputation variability. For instance, two predictors relating to poverty or inequality, Gini Index and Life Expectancy, both had low imputation variability despite Gini Index having the most missing values before MICE (about 67%) and life expectancy having only about 12% missing values.

Supplementary Table 1: Details on predictor variables used in clustering analysis of countries and territories that share a common model and in predictive modelling of wood fuel and wood charcoal. FAO = Food and Agriculture Organization of the United Nations; WHO = World Health Organization.

Variable Name	Definition	Units	Analytical Use	Source (date of access)	Website of Source<br>Notes on calculations and definitions
Population	Human population	1000 people	Underlying calculations	World Bank<br>And FAO	https://data.worldbank.org/indicator/SP.POP.TOTL
Rural Population	Human population living in rural areas	1000 people	Underlying calculations	World Bank	https://data.worldbank.org/indicator/SP.RUR.TOTL
Percent Urban	Percent of human population living in urban areas	%	Clustering, Predictive models		(Population-Rural population) / Population
Minimum Temperature	Monthly average temperature of the coldest month (1961 – 1999)	degrees C	Clustering, Predictive models	World Bank - Climate Change Knowledge Portal<br>(23/10/2020)	https://datacatalog.worldbank.org/dataset/climate-change-knowledge-portal-historical-data
Rainfall	Annual average rainfall (1961-1999)	mm	Clustering, Predictive models	World Bank - Climate Change Knowledge Portal<br>(23/10/2020)	https://datacatalog.worldbank.org/dataset/climate-change-knowledge-portal-historical-data
Forest Area		1000 hectare	Predictive models	FAO<br>(15/10/2020)	https://fra-data.fao.org/WO<br>Land spanning more than 0.5 hectares with trees higher than 5 meters and a canopy cover of more than 10 percent, or trees able to reach these thresholds in situ. It does not include land that is predominantly under agricultural or urban land use. Annual values based on linear interpolation.<br>Used as forest area per capita (Forest Area / Population).

Wooded Area		1000 hectare	Predictive models	FAO (15/10/2020)	https://fra-data.fao.org/WO<br>"Other wooded land" from the Global Forest Resource Assessment (FRA), defined as land not classified as "Forest", spanning more than 0.5 hectares; with trees higher than 5 meters and a canopy cover of 5-10 percent, or trees able to reach these thresholds in situ; or with a combined cover of shrubs, bushes and trees above 10 percent. It does not include land that is predominantly under agricultural or urban land use. Annual values based on linear interpolation.<br>Used as wooded area per capita (Wooded Area / Population).
Tree Area	Forested and wooded area	1000 hectare	Clustering		Forest Area + Wooded Area
Percent Trees	Percent of the country area forested or wooded	%	Clustering		(Forest Area + Wooded Area) / Total Area
GDP	Gross domestic product	US$ (current)	Underlying calculations	World Bank (14/08/2021)	https://data.worldbank.org/indicator/NY.GDP.MKTP.CD
GDP per capita	Gross domestic product per person	US$ (current) / person	Clustering, Predictive models		GDP / Population
Electricity	Proportion of people with access to electricity	%	Clustering	World Bank (08/06/2021)	https://data.worldbank.org/indicator/EG.ELC.ACCS.ZS
Life Expectancy	Life expectancy at birth	years	Clustering, Predictive models	World Bank (08/06/2021)	https://data.worldbank.org/indicator/SP.DYN.LE00.IN

Gini Index	Average Gini index, a measure of inequality, 1999-2020	0-100	Clustering, Predictive models	World Bank (08/06/2021)	https://data.worldbank.org/indicator/SI.POV.GINI?view=map
Number of Trees		tree	Clustering	Our World In Data (18/06/2021)	https://ourworldindata.org/forest-area Number of plants with woody stems larger than 10 cm diameter at breast height (DBH)
Biomass Use	Proportion of people that use energy from biomass for cooking	proportion	Predictive models	WHO[4,5]	https://www.who.int/data/gho/data/themes/topics/topic-details/GHO/household-air-pollution Estimates available at link above and derived from methods cited as WHO. Note that high income countries and territories* assumed to be 0.
Charcoal Use	Proportion of people that use energy from charcoal cooking	proportion	Predictive models	WHO[4,5]	https://www.who.int/data/gho/data/themes/topics/topic-details/GHO/household-air-pollution Estimates available at link above and derived from methods cited as WHO. Note that high income countries and territories* assumed to be 0.
Sawlogs	Sawlogs and veneer logs	m^3	Predictive models	FAO (11/08/2021)	http://www.fao.org/faostat/en/#data/FO Removals roundwood for manufacture of sawnwood, railway sleepers (ties), or veneer logs; includes both coniferous and non-coniferous.
OIR	Other industrial roundwood removals	m^3	Predictive models	FAO (11/08/2021)	http://www.fao.org/faostat/en/#data/FO Removals of roundwood for other industrial uses (not production of sawnwood, veneer, or pulpwood). It may be used for poles, piling, posts, fencing, pitprops, shingles and shakes, wood wool, tanning, distillation etc.

* Countries and territories are classified as high income by the World Bank for the 2024 fiscal year if they had a 2022 gross national income (GNI) per capita (calculated using the World Bank Atlas method) of $13,846 or above.

After the variables were fully imputed, we applied a smoothing procedure to time-varying predictors Biomass Use, Charcoal Use, Electricity, Forest Area, GDP, Gini Index, Life Expectancy, OIR, Population, Rural Population, Sawlogs, and Wooded Area. In smoothing these covariates, our aim was to capture the long-term signals in the mechanistic drivers of wood fuel and wood charcoal demand and filter out noise from interannual variability and measurement error. The smoothing procedure was applied (repeated) independently across variables and countries / territories. For a time series of a given predictor variable (which may include any mixture of original, interpolated, or imputed values), we assumed a generalized additive model[6] (GAM) with a single smooth penalized regression spline of time (year). The penalized regression splines were constructed using P-spline basis functions and a first-order smoothness penalty. We assumed a Gaussian distribution for all the smoothing GAMs, after applying a log transformation to Forest Area, GDP, OIR, Sawlogs, and Wooded Area. Predictions from the fitted GAMs, reversing the log transformation where applicable, are the final smoothed covariate inputs to the random forest regression models for wood fuel removals (modeled as wood fuel demand (consumption) per capita) and wood charcoal (modeled as a fraction of wood fuel demand).

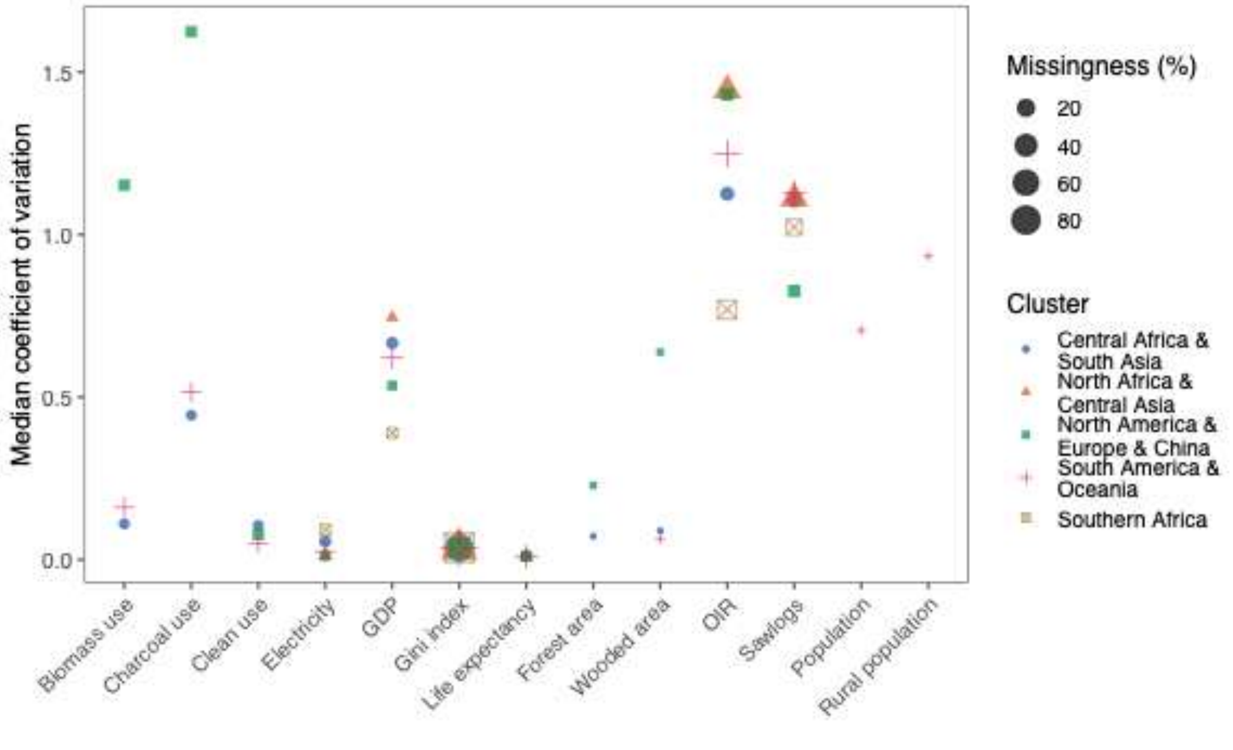

Supplementary Figure 1: Variability of imputed predictor variable values from the Multiple Imputation by Chained Equations (MICE) with Random Forests method. The coefficient of variation over the 1000 imputed data sets was computed for each imputed data point. Points on the y-axis are the medians over the coefficient of variation values for each variable (x-axis) and cluster (denoted by color and shape). Shape size represents the percentage of missing values after interpolation but prior to the MICE imputation.

Clustering of countries and territories

The clustering methodology is described in the main paper and variables used for clustering are identified in Supplementary Table 1. Here we indicate exactly which countries and territories are included in each cluster (Supplementary Table 2).

Supplementary Table 2: Countries and territories included inside each of the five clusters for modelling. The Russian Federation was identified as its own cluster and was not modelled. Analysis cluster name is the name used in the results and discussion of this paper. The cluster names are for simplicity in reporting; countries and territories in other areas are often included. First stage cluster refers to the clusters identified in the first stage of clustering analysis. In the second stage, a clustering analysis was applied to each of the first stage clusters. Andorra, Niue, Norfolk Island, and Saint Pierre and Miquelon were not assigned by the clustering analysis as there was not sufficient information. They were assigned manually based on surrounding countries and territories.

Analysis Cluster Name	First Stage Cluster	Countries and Territories in Analysis Cluster
Central Africa & South Asia	Africa & South Asia	Afghanistan, Bangladesh, Benin, Burkina Faso, Burundi, Central African Republic, Chad, Côte d'Ivoire, Djibouti, Eswatini, Ethiopia, Falkland Islands (Malvinas), Gambia, Ghana, Guinea, Guinea-Bissau, India, Lesotho, Madagascar, Malawi, Mali, Mauritania, Mayotte, Niger, Nigeria, Pakistan, Rwanda, Sierra Leone, Somalia, South Sudan, Sudan, Togo, Uganda, Yemen
Southern Africa	Africa & South Asia	Angola, Bhutan, Botswana, Cambodia, Cameroon, Comoros, Congo, Democratic People's Republic of Korea, Democratic Republic of the Congo, Eritrea, Haiti, Kenya, Kiribati, Lao People's Democratic Republic, Liberia, Micronesia (Federated States of), Mozambique, Myanmar, Namibia, Nepal, Papua New Guinea, Sao Tome and Principe, Senegal, Solomon Islands, South Africa, Timor-Leste, United Republic of Tanzania, Vanuatu, Zambia, Zimbabwe
North America & Europe & China	North America & Europe & North Africa & Central Asia	Albania, Andorra, Austria, Belarus, Belgium, Bosnia and Herzegovina, Bulgaria, Canada, China Hong Kong SAR, China Macao SAR, China mainland, China Taiwan Province of, Croatia, Cyprus, Czechia, Denmark, Estonia, Finland, France, Georgia, Germany, Greece, Hungary, Ireland, Italy, Japan, Latvia, Lebanon, Liechtenstein, Lithuania, Luxembourg, Montenegro, Netherlands, North Macedonia, Norway, Poland, Republic of Korea, Romania, Saint Pierre and Miquelon, Serbia, Slovakia, Slovenia, Spain, Sweden, Switzerland, United Kingdom of Great Britain and Northern Ireland, United States of America
North Africa & Central Asia	North America & Europe & North Africa & Central Asia	Algeria, Antigua and Barbuda, Armenia, Aruba, Azerbaijan, Bahrain, Barbados, Egypt, Grenada, Iran (Islamic Republic of), Iraq, Jordan, Kazakhstan, Kuwait, Kyrgyzstan, Libya, Maldives, Mongolia, Morocco, Oman, Qatar, Republic of Moldova, Saint Kitts and Nevis, Saint Lucia, Samoa, Saudi Arabia, Syrian Arab Republic, Tajikistan, Tonga, Tunisia, Turkey, Turkmenistan, Ukraine, United Arab Emirates, Uzbekistan
South America & Oceania	South America & Russian Federation & Oceania	Argentina, Australia, Bahamas, Belize, Bolivia (Plurinational State of), Brazil, British Virgin Islands, Brunei Darussalam, Cabo Verde, Chile, Colombia, Costa Rica, Cuba, Dominica, Dominican Republic, Ecuador, El Salvador, Equatorial Guinea, Fiji, French Guyana, French Polynesia, Gabon, Guadeloupe, Guatemala, Guyana, Honduras, Indonesia, Israel, Jamaica, Malaysia, Martinique, Mauritius, Mexico, New Caledonia, New Zealand, Nicaragua, Niue, Norfolk Island, Palau, Panama, Paraguay, Peru, Philippines, Portugal, Réunion, Saint Vincent and the Grenadines, Seychelles, Singapore, Sri Lanka, Suriname, Thailand, Trinidad and Tobago, Turks and Caicos Islands, Uruguay, Venezuela (Bolivarian Republic of), Viet Nam
Russian Federation	South America & Russian Federation & Oceania	Russian Federation

Protocol for external data search and data conversion

Data for model building were identified through a systematic data search as explained in the main paper. Here we describe the details of the data search protocol (Supplementary Table 3) and all conversion coefficients used in transforming those data into m^3 (wood fuel) and tonnes (charcoal) (Supplementary Table 4).

Supplementary Table 3: Protocol for external data search to identify existing data on wood fuel or wood charcoal production or consumption. Countries refers to both countries and territories. FAO = Food and Agriculture Organization of the United Nations.

Search type (Number of data points acquired)	Search details
Literature (274)	ScienceDirect using keywords: “woodfuel”, “wood fuel”, “fuelwood”, “fuel wood”, “firewood”, “charcoal”, “production”, and “consumption” and years 1999 – current year (2019 or 2020). After the first 20 countries, “NEAR” was used to refine searches.<br>For Spanish and French-speaking countries an additional search was conducted in Google Scholar using the title function and “leña, “madera”, “combustible de madera”, “carbón vegetal”, “carbón de leña”, “consumo”, “uso”, and “producción” (Spanish-speaking countries) and “bois énergie”, “bois de chauffage”, “dendroenergie”, “combustibles ligneux”, “bois a bruler”, “charbon de bois”, “bois de feu”, “consommation”, “usage”, “utilisation” and “production” (French-speaking countries).<br>Papers were excluded for the following reasons: data are estimates or model-based estimates (except energy balances), data are from FAO, data were impossible to scale to national level consumption or production, no data source cited, cited data source appears to be an estimate, or cited data source is available instead (in this case cited data source used even if not uncovered in original search).
Government websites (1856)	National websites for Departments or Ministries of: Energy, Renewable Energy, Statistics, Forestry, Environment, and Agriculture.<br>For Spanish- and French-speaking countries these terms were also searched in Spanish and French respectively. On each website, links and lists were searched for raw data, national surveys, reports from international organizations, statistical reports, yearbooks, and energy balances.
International Organizations, National Development Agencies, Non-governmental Organizations (179)	UN Development Program (UNDP), UN Environment Program (UNEP), Center for International Forestry Research (CIFOR), World Bank, US Agency for International Development (USAID), FAO, Department for International Development (DFID), Deutsche Gesellschaft für Internationale Zusammenarbeit (GIZ), Swedish International Development Cooperation Agency (Sida), UK aid were all originally search for reports publications or project data on wood fuel, wood charcoal, and firewood for each target country. International Institute for Environment and Development (IIED) was additionally searched for 15 relevant countries. These organizations were selected based on expert interviews.<br>Due to frequent references to wood fuel projects, Global Environmental Fund (GEF) and Energy Sector Management Assistance Program (ESMAP) were added on 30/10/2019. Resources did not allow these sites to be checked for countries previously searched.

Supplementary Table 4: Conversion coefficients used to transform all found data to m^3 (wood fuel) and tonnes (wood charcoal). (a) Proportion of coniferous and non-coniferous wood by country were estimated as proportion reported in FAOSTAT for production in 2018. All energy units were converted first to megajoules and then to m^3 (wood fuel) or metric tonnes (wood charcoal). MJ2m3 = megajoule to cubic meters = 1/9740. MJ2mt = megajoule to metric tonnes = 0.001/15.16. It is estimated that 6 m^3 wood fuel are required to make 1 tonne charcoal. In most cases, data sources tended to use tons and tonnes interchangeably and without seeming to use the spelling to distinguish British tons versus metric tonnes. We therefore assumed metric tonnes (1000 kg) unless it could be clearly determined that tons was meant to imply British tons (1016 kg) or American tons (907 kg). Conversion factors were obtained from multiple sources[7-11]. Here "w2v" are FAO weight (kg) to volume (m^3) conversions[10] given in (b).

(a)

Units	Type of unit	Conversion factor<br>Wood fuel (m^3)	Charcoal (tonnes)
1000 tonnes ('000 tons)	Mass	1000*1000*w2v	1000
cubic feet	Volume	2.83/100	(2.83/100)*(1/6)
grams (g)	Mass	0.001*w2v	0.000001
gigjoule (GJ)	Energy	1000*MJ2m3	100*MJ2mt
1000 barrels of oil equivalent (kboe)	Energy	6118*1000*MJ2m3	6118*1000*MJ2mt
kilograms oil equivalent (kgoe)	Energy	41.868*MJ2m3	41.868*MJ2mt
kilograms (kg)	Mass	w2v	0.001
1000 tonnes of oil equivalent (ktoe)	Energy	41868*1000*MJ2m3	41868*1000*MJ2mt
kilowatt hours (kWh)	Energy	3.6*MJ2m3	3.6*MJ2mt
m^3	Volume	1	1/6
m^3 overbark	Volume	88/100	(88/100)*(1/6)
m^3 roundwood equivalent	Volume	1	1/6
m^3 wood equivalent	Volume	99/100	(99/100)*(1/6)
metric tonnes / metric tons	Mass	1000*w2v	1
petajoule (PJ)	Energy	1,000,000,000*MJ2m3	1,000,000,000*MJ2mt
petajoule lower heating value (PJ LHV)	Energy	1,000,000,000*MJ2m3	1,000,000,000*MJ2mt
quintal	Mass	100*w2v	0.1
Stere	Volume	66/100	(66/100)*(1/6)
terajoules (TJ)	Energy	1,000,000*MJ2m3	1,000,000*MJ2mt
tonnes of oil equivalent (toe)	Energy	41868*MJ2m3	41868*MJ2mt
tons (US tons)	Mass	907*w2v	0.907
tons of coal equivalent (tce)	Energy	29,308*MJ2m3	29,308*MJ2mt
terawatt hours (TWh)	Energy	3,600,000,000*MJ2m3	3,600,000,000*MJ2mt
terawatt hours for projects of common interest (TWh PCI)	Energy	3,600,000,000*MJ2m3	3,600,000,000*MJ2mt

(b)

	European	Non-European
Coniferous	1/410	1/476
Non-coniferous	1/557	1/597

Compilation of response variables and transformations for wood fuel and charcoal

To create the response variables for modeling, we used the strongest available FAOSTAT data (as described in “Input data on wood fuel production from FAO”) on wood fuel removals for countries / territories and years where it was available (N = 1025 for wood fuel, N=567 for charcoal). We filled in values that were unavailable with data found from the literature search (Supplementary Table 3), where available (N = 1409 for wood fuel, N=1144 for charcoal). For the retained FAOSTAT data on wood fuel and wood charcoal, we estimated apparent consumption, hereafter demand, as production plus imports less exports.

To create the response variable for wood fuel, we calculated wood fuel demand per capita as wood fuel demand divided by Population (Supplementary Table 1) for the given year and country. To create the response variable for charcoal, we computed the fraction of wood energy demand met with wood charcoal as the demand for wood charcoal divided by the demand for wood fuel. For the retained FAOSTAT data, we divided charcoal demand by the wood fuel demand values from the retained FAOSTAT data. Where those wood fuel values were missing, we used predicted wood fuel demand from the wood fuel random forests. For the found data, we divided charcoal demand by the predicted wood fuel demand from the random forest models. Where the resulting fraction of wood energy demand met with wood charcoal was less than 0% or greater than 100%, we replaced the value with 0% or 100%, respectively.

The values of per capita demand for wood fuel have a heavy-tailed distribution and we applied a log transformation for modelling, resulting in a distribution closer to a Gaussian distribution. Fit and tuning of the random forests were therefore optimized for prediction performance on the log scale. We modelled the charcoal fraction at the logit scale.

For 17 countries or territories, missing export or import data in FAOSTAT in 2017, 2018, and 2019 limited the number of available observations for model-building. For these countries and territories, trade data were explored in UN Comtrade (https://comtradeplus.un.org/), freely available global trade data, and also as raw data and mirror data in the Trade Data Monitor (TDM), a subscription source of trade data for 118 countries and territories. For all but three of these countries / territories, trade data were either not reported or minimal (i.e., valued at less than $500,000 USD in 2 of the 3 years). For the three countries, we added the new data found. These included import data from UN Comtrade for The Gambia, mirrored estimates of export from TDM for Cuba, and estimates of export, which were the same in both UN Comtrade and TDM for El Salvador. Other trade data for these 17 countries / territories and three years were assumed to be 0 to enable maximal use of available information on wood fuel removals and wood charcoal production.

Supplementary Methods 2: Model Evaluation

Exploration of model composition

In addition to the variable permutation importance scores for the wood fuel demand per capita and fraction of demand met with charcoal models (Figure 6), we also evaluated variable use frequency (Supplementary Figures 2 and 3). Variable use frequency is an additional tool for summarizing the composition of all the decision trees that make up a random forest model. This information is complementary to that of Figure 6 and was used as part of our overall assessment of the composition of each input variable to modeled estimates. Predictor variables are defined in Supplementary Table 1.

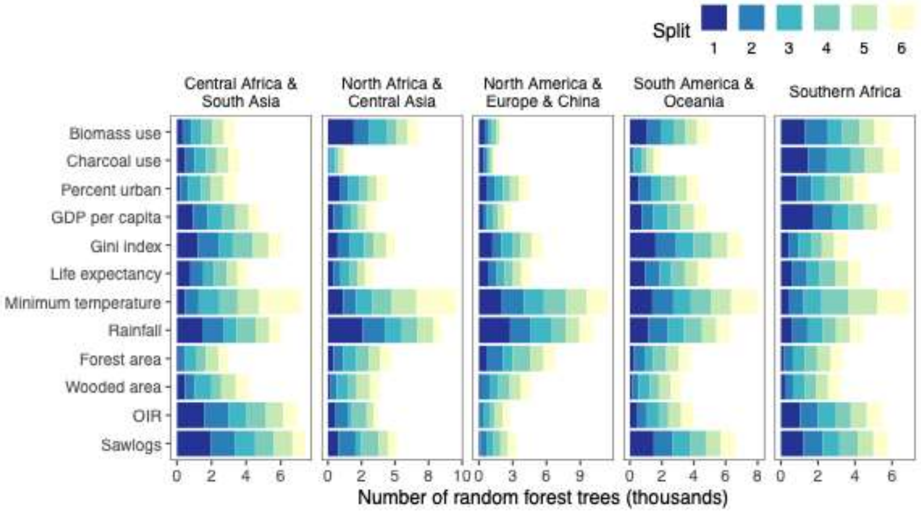

Supplementary Figure 2: Variable use frequency for splits 1 to 6 in the wood fuel demand models, i.e., the number of random forest trees (thousands) for which a variable appears in each of the first 6 splits.

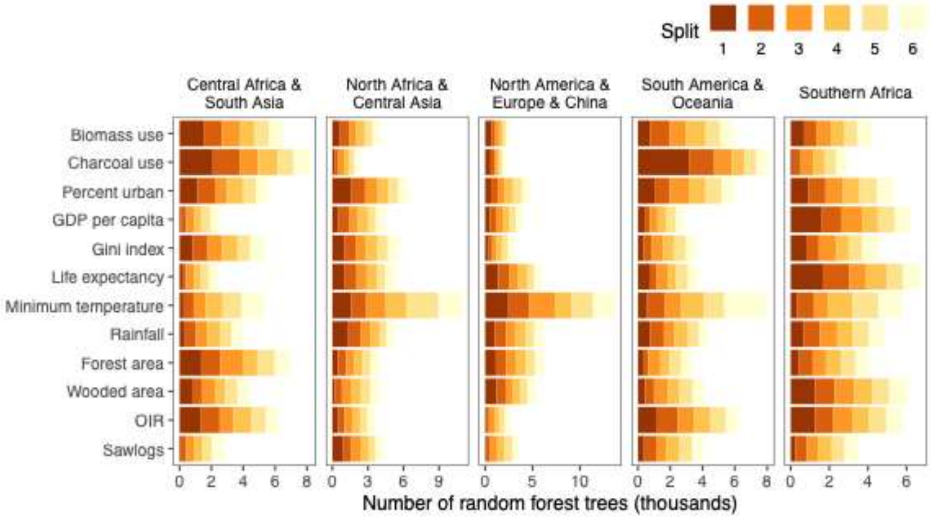

Supplementary Figure 3: Variable use frequency for splits 1 to 6 in the charcoal fraction models, i.e., the number of random forest trees (thousands) for which a variable appears in each of the first 6 splits.

Methods for checks of model fit

We assessed both in-sample and out-of-sample prediction accuracy for each of the models. In-sample model fit describes the ability of the models to capture per capita wood fuel demand and charcoal fraction values included in the models as data. For in-sample model fit, we compared the observed per capita wood fuel demand values and charcoal fraction values from both the retained FAOSTAT and found data to corresponding fitted values (i.e., predictions) from the final random-forest models.

Out-of-sample prediction performance describes the ability of the models to predict per capita wood fuel demand and charcoal fraction values that have not been seen by the model as data. To assess this, we carried out a 10-fold cross-validation procedure separately for each cluster. We randomly split the data for a given cluster into 10 partitions, which represented 10 sets of "test" data. For each of the 10 test data sets, we held back data from the test set, fit the model to the remaining data (i.e., training data), and used the fitted random forest to predict the per capita values and charcoal values in the test data. The whole 10-fold cross-validation procedure was repeated 10 times, resulting in 10 out-of-sample predictions for every per capita wood fuel demand value and charcoal fraction value in the data set.

Finally, we quantified accuracy by computing mean absolute errors for both in-sample and out-of-sample predictions (including all repetitions of the 10-fold cross-validation), i.e., the mean absolute difference between the predicted and observed values. To further quantify in-sample fit, we computed the squared Pearson correlation coefficients (r^2) between the predicted and observed values. For out-of-sample fit, we computed an out-of-sample version[12] of r^2.

Results for checks of model fit for wood fuel production

Supplementary Figure 4 shows scatter plots of fitted values for wood fuel demand per capita versus the original data. The models are broadly more accurate for in-sample retained FAOSTAT data than for found data, reflecting the greater variance among the found data. The fitted values generally capture the in-sample data closely, however, there is some under-prediction of the found data among the highest values. Most noticeably, the model for the South America & Oceania cluster under-predicts a collection of found data points for Venezuela exceeding $2m^3$ of wood fuel demand per capita.

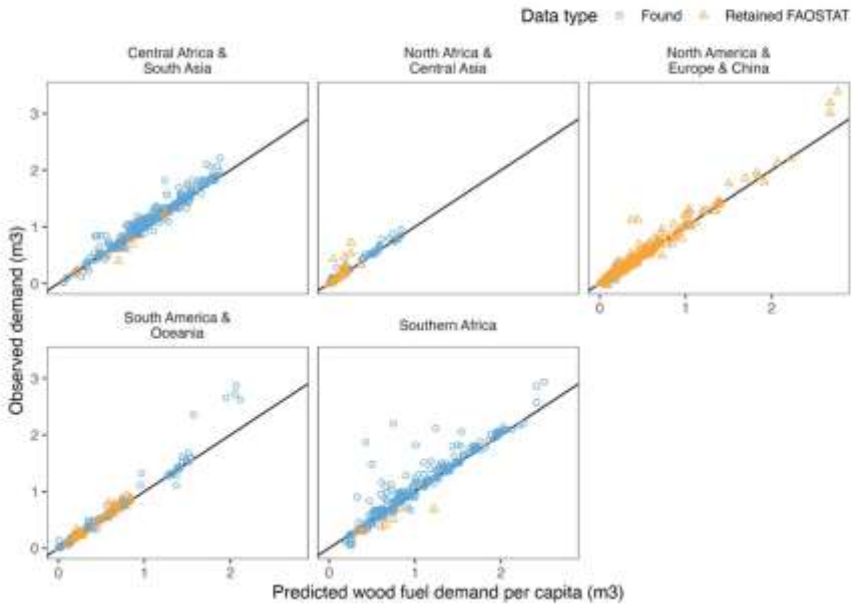

Supplementary Figure 4: Scatter plots assessing in-sample model predictions for wood fuel demand per capita. Observed values on the y-axis are the original values from the retained FAOSTAT data (orange triangle) and found data (blue circles). Fitted values on the x-axis are from the random forest models.

Mean in-sample prediction errors for wood fuel were lowest in the North Africa & Central Asia cluster at $0.021m^3$ per capita and were highest in Southern Africa at $0.085m^3$ per capita (Supplementary Figure 4). The corresponding in-sample r^2 values ranged from 0.897 (Southern Africa) up to 0.969 in North America & Europe & China (where $r^2=1$ would indicate perfect fit). Mean absolute errors for the out-of-sample predictions (Supplementary Figure 5) were naturally higher than the associated in-sample mean errors, ranging from $0.039m^3$ per capita in the North Africa & Central Asia cluster to $0.201m^3$ per capita for Southern Africa.

Supplementary Table 5: Prediction accuracy measures of the random forest models for wood fuel demand per capita and the fraction of wood fuel demand met by charcoal. The table presents the mean absolute error and coefficient of determination (r^2) for in-sample predicted values (In) and out-of-sample predicted values from the repeated 10-fold cross validation experiment (Out). An out-of-sample version[12] of r^2 is used for the latter.

	Wood fuel				Charcoal			
	Mean absolute error (m^3)		r^2		Mean absolute error (pp)		r^2	
	**In**	**Out**	**In**	**Out**	**In**	**Out**	**In**	**Out**
Central Africa & South Asia	0.050	0.186	0.950	0.454	1.55	4.84	0.964	0.742
North Africa & Central Asia	0.021	0.039	0.925	0.811	4.46	13.28	0.889	0.182
North America & Europe & China	0.026	0.047	0.969	0.906	2.11	4.24	0.967	0.861
South America & Oceania	0.026	0.051	0.959	0.857	3.31	6.38	0.943	0.788
Southern Africa	0.085	0.201	0.897	0.606	2.41	6.72	0.934	0.622

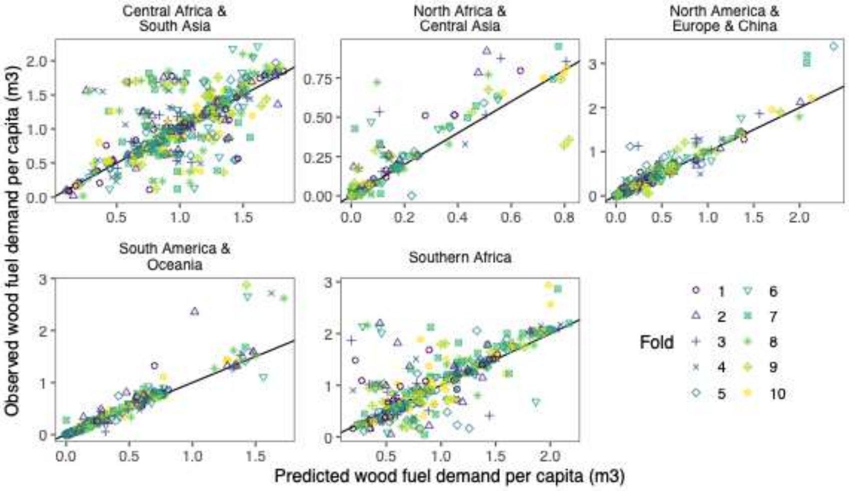

Supplementary Figure 5: Scatter plots assessing out-of-sample model predictions for wood fuel demand per capita. Observed values on the y-axis are the original values from the data that were held out from model training during repetition 1 of the 10-fold cross-validation. Predicted values on the x-axis are from the random forest models. Shape and color indicate which of the 10 out-of-sample test sets each data point belonged to in repetition 1.

Results of checks of model fit for charcoal fraction

Supplementary Figure 6 shows scatter plots of predicted versus in-sample data values of the fraction of wood fuel demand met by charcoal. There is generally a strong correspondence between the in-sample data and the predictions, with only a few sizeable model errors.

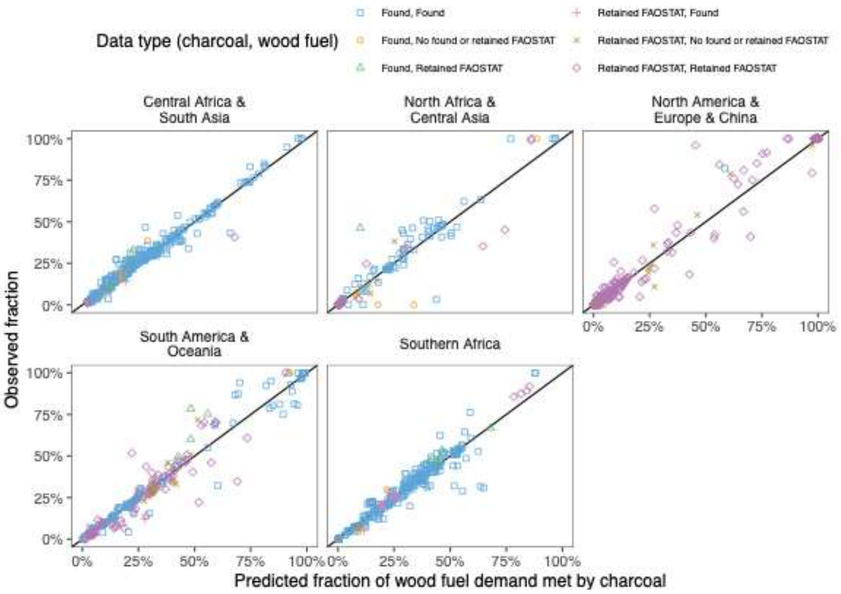

Supplementary Figure 6: Scatter plots assessing in-sample model predictions for the fraction of wood fuel demand met by charcoal. Observed values in the y-axis are the original fractions from retained FAOSTAT and found data sources: the shape and color of the points reflect the data source combinations for the numerator (charcoal demand) and denominator (wood fuel demand) of the fraction. Predicted values in the x-axis are from the random forests.

Mean in-sample prediction errors for the charcoal fraction (Supplementary Figure 6) range from 1.55 percentage points (pp) for the Central Africa & South Asia cluster to 4.46 pp (North Africa & Central

Asia). Meanwhile, the in-sample r^2 values were high, the lowest being 0.889 (North Africa & Central Asia) and reaching 0.967 for North America & Europe & China. The corresponding mean absolute errors for the out-of-sample predictions (Supplementary Figure 7) range from 4.24 pp (North America & Europe & China) up to 13.28 pp (North Africa & Central Asia). The latter had the weakest out-of-sample r^2 value at 0.182, whereas the r^2 values for the other four clusters were reasonably high, ranging from 0.622 (Southern Africa) to 0.861 (North America & Europe & China).

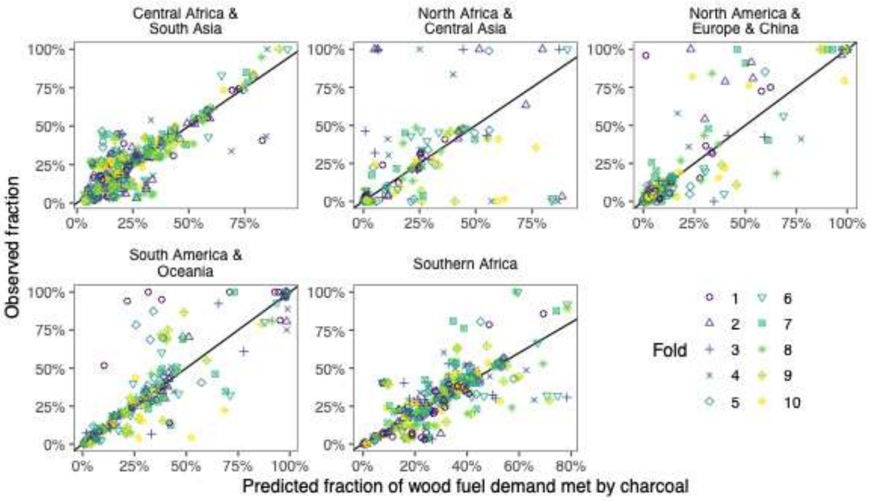

Supplementary Figure 7: Scatter plots assessing out-of-sample model predictions for the fraction of wood fuel demand met by charcoal. Observed values on the y-axis are the original values from the data that were held out from model training during repetition 1 of the 10-fold cross-validation. Predicted values on the x-axis are from the random forest models. Shape and color indicate which of the 10 out-of-sample test sets each data point belonged to in repetition 1.

Supplementary References

[1] IEA, IRENA, UNSD, World Bank & WHO. *Tracking SDG7: The Energy Progress Report*. World Bank, Washington DC, https://sdgs.un.org/publications/tracking-sdg7-energy-progress-report-2024-58022 (2024).

[2] United Nations Statistics Division. Standard Country or Area Codes for Statistical Use (M49), https://unstats.un.org/unsd/methodology/m49 (2024).

[3] Wilson, S. *miceRanger—fast imputation with random forests in R*. R package version 1.5.0, https://CRAN.R-project.org/package=miceRanger (2021).

[4] Stoner, O. et al. Household cooking fuel estimates at global and country level for 1990 to 2030. *Nature Communications* **12**, 5793 (2021).

[5] Stoner, O. et al. Global household energy model: a multivariate hierarchical approach to estimating trends in the use of polluting and clean fuels for cooking. *Journal of the Royal Statistical Society: Series C (Applied Statistics)* **69**, 815-839 (2020).

[6] Wood, S. N. *Generalized Additive Models: An Introduction with R*, Second Edition. (CRC Press, 2017).

[7] United Nations. 2013 *Energy Balances*, https://www.un-ilibrary.org/content/books/9789210577779 (2015).

[8] Glasenapp, S., Fonseca, M., Weimar, H., Döring, P. & Aguilar, F. X. Conversion factors for residential wood energy in the European Union: an introduction to harmonizing units of measurement. *Renewable and Sustainable Energy Reviews* **138**, 110491 (2021).

[9] FAO, ITTO & United Nations. *Forest product conversion factors*, https://openknowledge.fao.org/items/2ccf10c6-6e0c-4d5f-a4df-82a910279bd9 (2020).

[10] IOR Energy Pty Ltd. *List of common conversion factors (Engineering conversion factors)*, https://w.astro.berkeley.edu/~wright/fuel_energy.html (2024).

[11] Gershtein, S. & Gershtein, A. *Instant Energy and Work Units Conversion. Energy Converter. Coal Energy Equivalent*, https://www.convert-me.com/en/convert/energy/tce.html?u=tce&v=1 (2024).

[12] Hawinkel, S., Waegeman, W. & Maere, S. Out-of-Sample R2: Estimation and Inference. *The American Statistician*, 1-11 (2024).
