# Supplementary material for: Global wood fuel production estimates and implications
Source: Nat Commun. 2025 Jul 15;16:6227. doi: 10.1038/s41467-025-59733-y (PMC12264136; doi:10.1038/s41467-025-59733-y)
Supplement: Supplementary file 2 — Description of Additional Supplementary Files [file 41467_2025_59733_MOESM2_ESM.pdf]

Description of Additional Supplementary Files

Supplementary Data 1. Country-level modelled estimates of total removals from forests and trees outside forests for wood fuel (Wood fuel) and estimates of charcoal production (Charcoal).

Supplementary Data 2. Continent-level modelled estimates of total removals from forests and trees outside forests for wood fuel (Wood fuel) and estimates of charcoal production (Charcoal).

Supplementary Data 3. Found data from the external data search, transformed and scaled for use in modelling.

Supplementary Data 4. FAOSTAT data (where available) for countries for which we did not produce modelled estim
